# Migraine and episodic Vertigo: a cohort survey study of their relationship

**DOI:** 10.1186/s10194-019-0991-2

**Published:** 2019-04-08

**Authors:** Christian Lampl, Alan Rapoport, Moris Levin, Elisabeth Bräutigam

**Affiliations:** 1 Headache Medical Centre, Linz, Ordensklinikum Linz Barmherzige Schwestern, 4020 Linz, Austria; 2 Department of Neurology, David Geffen School of Medicine at UCLA, Los Angeles, CA USA; 3 Department of Neurology, University of California San Francisco, San Francisco, CA USA; 4 Department of Radio-Oncology Ordensklinikum Linz Barmherzige Schwestern, Linz, Austria

**Keywords:** Migraine, Vertigo, Episodic vertigo, Vestibular migraine

## Abstract

**Background and aim:**

Migraine headache and vestibular-type vertigo co-occur in the general population about three times more often than expected by chance. Attacks of episodic vertigo (eV) are currently not recognized as migraine equivalents or variants in the International Classification of Headache Disorders, 3rd edition (ICHD III). No strong data exist about the prevalence of eV during the phases of a migraine attack. The aim of this study is to analyze the timing association between migraine-related episodic vertigo and the phases of migraine.

**Methods:**

The “Migraine and Neck Pain Study” gathered data from nearly 500 adult participants in a questionnaire-based survey. In this prospective, follow-up study we re-analyzed patients with episodic migraine with and without aura who experienced eV anytime around their migraine attacks. For this we defined 3 different time periods.

**Results:**

146/487 (30%) reported eV anytime during the migraine attack; 79/487 (16%) that noticed eV with the start of the headache, 51/487 (10%) within 2 h before the headache and 16/487 (3%) experienced eV 2–48 h before the headache, as a premonitory symptom. 130/487 (26.7%) of our patients can be diagnosed with vestibular or probable vestibular migraine supporting the clinical association of migraine and vertigo.

**Conclusions:**

Our results seem to further support the concept that vertigo in migraine is best thought of as an integral manifestation of migraine, rather than a prodromal or aura symptom.

## Introduction

Migraine is very prevalent worldwide, with population-based studies reporting migraine in 4–8% of men and 11.2–18.2% of women in both the US [1] and Europe [2]. Migraine headache and co-occur in the general population about three times more often than expected by chance: at a lifetime migraine prevalence of 16% [3] and vertigo of 7% [4], concurrence was actually found in 3.2% of the population [5]. This demonstrates the need for further evaluation of vertigo in migraineurs. One of the major problems is nomenclature, due to the overlap in clinical features between different terminologies of vertigo and their association with migraine (vestibular migraine, migraine-associated episodic vertigo, migraine-associated dizziness, migraine-related vertigo, benign recurrent vertigo and migraine-related vestibulopathy). We need clarification of the terms used to describe various combinations of symptoms, and information about which ones occur at which time within a migraine phase. Slater first introduced the term benign recurrent vertigo (BRV) [6] to describe spontaneous attacks of episodic vertigo (eV) not explained by either central or otological abnormalities, which did not lead to permanent deficits. BRV and eV are currently not recognized as migraine equivalents or variants in the International Classification of Headache Disorders, 3rd edition (ICHD III), although there is a defined condition Vestibular Migraine, in the Appendix (1.6.6) [7]. In contrast, benign paroxysmal vertigo (BPV) of childhood is recognized as one of the periodic syndromes that precede the development of migraine. To our knowledge, no strong data exist about the prevalence of eV during the different phases of a migraine attack. Therefore the timing association between eV and the phases of migraine remains unclear.

The “Migraine and Neck Pain Study” [8] gathered data from nearly 500 randomly-selected adult participants in a questionnaire-based survey, conducted in Austria and Greece. The survey included time of onset, side and quality of neck pain in migraine patients, as well as patients’ associated migraine symptoms (sensitivity to light, sound and smell, nausea, vomiting and aggravation of pain by physical activity) and the concurrence of eV. We have re-analysed the data from this study regarding the timing association between eV and migraine during different phases. We hypothesized a high prevalence of eV during the different phases of a migraine attack.

## Methods

The methods of the “Migraine and Neck Pain Study” have been described in detail elsewhere^8^ and are briefly summarized here for the eV survey. The definition used for eV (according to the definition of episodic recurrent vertigo, or ERV) was spontaneous attacks of vertigo not explained by either central or otological abnormalities, that did not lead to permanent deficits. Eligible patients with episodic migraine with aura (MwA) and/or without aura (MwoA), diagnosed by neurologists, were asked in detail if they experienced eV anytime around their migraine attacks. The migraine was divided into 3 time segments: onset of headache; < 2 h before the onset of headache and 2–48 h before the onset of headache. We tried to answer the question of whether eV is a prodromal symptom, or part of the headache phase of migraine. At the time of inclusion of our patients, the ICHD-III beta [9] was used. Herein prodromal (premonitory) symptoms were defined as “symptoms preceding, and forewarning of, a migraine attack by 2-48 hours, occurring before the aura in MwA and before the onset of pain in MwoA”. Hence, for our analyses, eV as prodromal (premonitory) symptom was defined according to this definition.

Inclusion criteria included men or women, aged 18–65 years, with MwA and/or MwoA, pre-diagnosed by a neurologist according to the definition of the International Classification of Headache Disorders 3 beta version [9]. Patients with known or suspected cervicogenic headache, history of significant cervical trauma or surgery, fibromyalgia and any general pain syndrome were excluded.

The study conformed to the revised ethical principles of the Helsinki declaration and the Codex rules and guidelines for research [10, 11].

All patients who reported eV at any time during the migraine attack were allocated to 3 period-groups as follows:

A. = consistently eV and any associated migraine symptoms at time of onset of headache;
B. = consistenly eV and any associated migraine symptoms starting < 2 h before the aura in MwA and before the onset of pain in MwoA;
C. = eV (independent of their frequency) any and associated migraine symptoms starting 2–48 h before the onset of headache. All questions ideally were answered immediately after the migraine attack, but no longer than 6 h after the end of the attack.

If eV consistently occurred in the time period A and B, it was interpreted as part of the migraine phase itself.

### Statistical analysis

We used Statistical Package for Social Studies (SPSS) version 17.0 (Aug 23, 2008) (SPSS Inc., Chicago, IL) to analyse our data. In most cases these were of participants´ response s to the various questions, summarized for all those with migraine. We described categorical variables as proportions (n [%]) and continuous variables in terms of means and standard deviations (SD). We assumed that eV is more prominent during the migraine headache phase. No statistical power calculation was conducted prior to the study. The sample size was based on the available data. Wilcoxon Signed Ranks tests were used for comparisons between patients with and without aura, a statistically significant result set at the 95% level (*p* = 0.05).

## Results

500 questionnaires were randomly distributed in the headache centres of Linz and Athens. 13 questionnaires had to be excluded from the analysis due to lost or missing data. The total study population (*n* = 487) consists of 356 females [73.1%] and 131 male [26.9%]). Mean age was 38 years (range, 19 to 61 years); mean age at onset of migraine was 21 years (range, 11 to 42 years). The median number of days of migraine attacks during the study period was 6 per month (range 3–14). 146/487 (30%) patients reported eV anytime during the migraine attack, 79/487 (16.2%) patients noticed eV with the start of the headache phase (group A); 51/487(10.5%) patients reported eV within 2 h before the headache phase (group B) and progressed into the headache phase in all patients; 16/487 (3.3%) patients experienced eV 2–48 h before the headache phase (group C) and progressed into the headache phase in 3 patients. We found that 375/487 (77%) patients had MwoA and 112/487 (22.9%) patients had MwA.

**Fig. 1:**
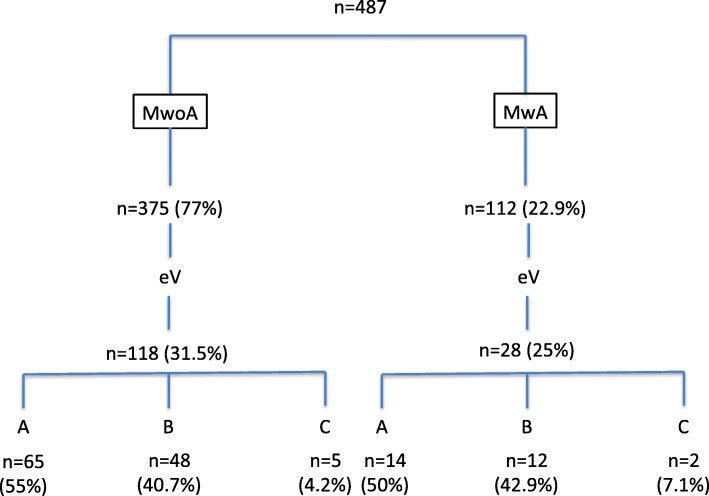
Flowchart of patients enrolled in the study with a breakdown into migraine subtypes and the composition of the 3 classes in each

Figure 1 is a flowchart of patients enrolled in the study with a breakdown into migraine subtypes and the composition of the 3 classes each. Table 1 shows number of migraine-associated symptoms in different groups. In group A, all patients showed typical migraine associated symptoms: 82.5% had an aggravation of headache pain during physical activity, 92.6% had nausea, 57.1% were sensitive to smell, 77.6% showed sensitiveness to sound and 82.3% to light. Neck pain was present in 37.7%; In group B, 7.6% observed headache aggravation during physical activity, 22% had nausea, 6.8% were sensitive to smell, 12.7% showed sensitiveness to sound and 16.2% to light. In this group neck pain was reported in 24.2%. In group C 1.4% had nausea, 0.6% were sensitive to smell, 1.6% to sound and 0.6% to light and neck pain was reported in 7.4%.

**Table 1:** Number of migraine associated symptoms in different groups (*n* = 487)

group	A	B	C
episodic vertigo	79 (16.2%)	51 (10.5%)	16 (3.3%)
aggravating headache pain by physical activity	402 (82.5%)	37 /7.6%)	0 (o%)
vomiting	189 (55.9%)	8 (1.6%)	0 (0%)
nausea	451 (92.6%)	107 (22.0%)	7 (1.4%)
sensitivity to smell	278 (57.1%)	33 (6.8%)	3 (0.6%)
sensitivity to sound	378 (77.6%)	62 (12.7%)	8 (1.6%)
sensitivity to light	401 (82.3%)	79 (16.2%)	2 (0.6%)
neck pain bilateral	59 (12.1%)	41 (8.4%)	3 (0.6%)
neck pain unilateral	125 (25.6%)	77 (15.8%)	33 (6.8%)

Table 2 show an evaluation of all patients with eV (*n* = 146) and their associated symptoms per group. We found a high association between the co-occurrence of eV and neck pain (*p* = .015), seen in all three groups.

**Table 2:** Number of patients in different groups with migraine-associated eV and their specific associated symptoms (n = 146)

group	A (*n* = 79)	B (*n* = 51)	C (*n* = 16)
aggravating pain by physical activity	79 (34.2%)	0 (0%)	0 (0%)
vomiting	19 (24.0%)	3 (5.9%)	0 (0%)
nausea	39 (49.4%)	17 (33.4%)	2 (12.5%)
sensitivity to smell	28 (35.4%)	3 (5.9%)	0 (0%)
sensitivity to sound	54 (68.4%)	3 (5.9%)	0 (0%)
sensitivity to light	47 (59.5%)	8 (15.7%)	2 (12.5%)
neck pain bilateral	38 (48.1%)	34 (66.7%)	13 (81.2%)
neck pain unilateral	25 (31.6%)	7 (13.7%)	0 (0%)

## Discussion

In this prospective, follow-up study of a cohort of patients with episodic migraine, 30% reported eV anytime during the migraine attack; there were 16% that noticed eV with the start of the headache, 10% reported eV within 2 h before the headache and 3% experienced eV 2–48 h before the migraine headache started. Similar to our results, previous studies showed that vertigo occurred in 24%–38% of migraine patients [12–14]. With respect to our definition of a prodromal state (2–48 h before headache), only 3% of migraine patients experienced eV as a symptom in this phase, suggesting it is more of a headache phase phenomenon, rather than prodromal.

In all 3 groups of subjects with associated vertigo, neck pain was highly prevalent. Interestingly, MwoA and MwA subjects differed very little in 1) the prevalence of associated vertigo (31.5% v. 25%) and 2) the timing of eV (with a nearly identical distribution into groups A, B and C). This would seem to further support the concept that vertigo in migraine is best thought of as an integral manifestation of migraine, rather than a prodromal or aura symptom.

In the new classification of headache disorders, ICHD III [8], the term ‘prodrome’, has now replaced ‘premonitory phase’ or ‘premonitory symptoms’ and does not include aura. Prodromal symptoms may begin hours or a day or two before the other symptoms of a migraine attack with aura. This is in contrast to the older classification ICHD III beta, where premonitory symptoms were given a more or less exact time period (symptoms preceding and forewarning of a migraine attack by 2–48 h). This reduced specificity highlights the lack of clear understanding of the prodrome itself.

Several studies have investigated the association of vertigo and other vestibular symptoms in migraineurs [15]. However the new ICHD III classification does not include a comprehensive category for these disorders. Only BPV of childhood is recognized as a distinct entity of the IHS migraine classification in childhood, but does not apply to our patients in whom eV undoubtedly started in adulthood. Similarly, the older designation basilar migraine (now termed migraine with brainstem aura) does not apply to our patients with eV because this diagnosis requires at least two symptoms from the posterior circulation territory, each lasting between 5 and 60 min.

Although we did not study eV separated in time from a migraine attack (48 h before, or clearly occurring sometime after the attack), we suspect such episodes may be attacks of vestibular migraine.

ICHD III describes the term vestibular migraine (VM) in the Appendix (1.6.6) and is now the somewhat accepted name for vestibular symptoms that are causally related to migraine [7]. Per this appendix definition, vestibular symptoms should be of moderate or severe intensity, lasting between 5 min and 72 h and half of the episodes should be associated with at least one of 3 clinical features associated with migraine including headache, phonophobia and photophobia and aura. In our cohort we found photophobia (16.6%) and phonophobia (14.3%) as accompanying symptoms. As per definition, one symptom is sufficient during a single episode (whereas different symptoms may occur during different episodes). We therefore can conclude that nearly 26% of our patients can be diagnosed with VM (or at least probable VM), supporting the clinical association of migraine and vertigo. Results of a tertiary vertigo centre found similar results, with 20.2% of their patients having VM [16].

A strength of our study is the high participation rate, indicating that the study cohort represents a migraine population with little selection bias. Also, we obtained reliable documentation for the timing of symptoms. Limitations may be that we did not use an e-diary. As eV was determined via questionnaire, we were not able to perform physical examinations and measurements. Further, we cannot exclude that some of these eV episodes are an expression of non-vertiginous “dizziness” (since there was no face to face interview), or other possible conditions that could mimic it. For example, in a migraineur who suffered a migraine episode following an attack of BPPV, the positional vertigo would not be considered part of the migraine episode, but the trigger for it. There may be also an overlap between Meniere’s disease and migraine, and even more between vestibular migraine and Meniere’s; attacks of Meniere’s disease are known to trigger migraine headaches - again, in cases like this, the vertigo would be considered a migraine trigger. Of note, is the fact that in the vestibular migraine Appendix definition in ICHD III, head motion-induced dizziness is the only acceptable form of dizziness as a vestibular symptom.

Approximately one quarter of our patients showed a fixed association of headache and vertigo, which is in agreement with previous reports [12, 17, 18].

The association of eV and migraine may result from various pathophysiological mechanisms. No studies have been carried out of the possible causal association of eV and migraine, neither is it known whether a reciprocal association occurs between eV and migraine. Parallel activation of vestibular and cranial nociceptive pathways [19–22] may lead to prolonged nociceptive activity, some perhaps involving cervical systems, that could lead to continuous afferent signalling of the nucleus caudalis, and, hence, activation of the trigeminovascular system. As the pathogenesis of migraine is linked to the trigeminal innervations of the cranial blood vessels, noxious stimuli from the inner ear and cervical structures may also play a role in this pathogenesis by facilitating central sensitization [23]. The mechanisms underlying vestibular dysfunction concurrent with migraine headache or separated from the headache phase still need further clarification, and future research is in order. Our next trial would be prospective, utilizing an electronic calendar and include a semi-structured interview aimed at more detailed characterization of the array of vestibular symptoms and careful recording of all aspects of vertiginous episodes outside of the migraine attack, as well as timing during attack.

## Conclusion

The symptom of eV is more of a headache phase phenomenon, rather than prodromal. We hope that elucidating the relative paucity of eV during this phase might eventually perhaps shed more light on this fascinating and to patients, distressing, phase of the migraine attack. We expect future studies that focus on the character and duration of vertigo in migraineurs and their variables outside of migraine attacks, will discover that many of these episodes will satisfy criteria for the diagnosis of vestibular migraine.

## Abbreviations

BPV: Benign paroxysmal vertigo
BRV: Benign recurrent vertigo
ERV: Episodic recurrent vertigo
eV: Episodic vertigo
ICHD III: International Classification of Headache Disorders, 3rd edition
MwA: Migraine with aura
MwoA: Migraine without aura
SD: Standard deviations
SPSS: Statistical Package for Social Studies
VM: Vestibular migraine
